# Beyond Recanalization: Neurovascular Unit Protection and Precision Adjunctive Therapy for Ischemic Stroke in the Reperfusion Era

**DOI:** 10.3390/biom16091346

**Published:** 2026-09-16

**Authors:** Xusheng Wu, Yue Xing, Jing Ma, Xianhui Wang, Xingjiang Xiong

**Affiliations:** 1 Clinical Medical College, Qinghai University, Xining 810016, China; 2 Department of Traditional Chinese Medicine, Medical College, Qinghai University, Xining 810001, China; 3 Department of Cardiology, Guang’anmen Hospital, China Academy of Chinese Medical Sciences, Beijing 100053, China

**Keywords:** acute ischemic stroke, reperfusion, neurovascular unit, cerebroprotection, no-reflow, blood–brain barrier, precision medicine, thrombectomy

## Abstract

Intravenous thrombolysis and endovascular thrombectomy (EVT) have increased large-vessel recanalization in acute ischemic stroke, yet macrovascular reopening does not ensure tissue reperfusion or functional recovery. Microvascular no-reflow, blood–brain barrier failure, oxidative injury, thromboinflammation, edema, and hemorrhagic transformation sustain a recanalization-to-recovery gap. Classical neuroprotection failed because of mismatches in models, timing, brain exposure, patient selection, and endpoints, and because ischemic injury was reduced to a single neuronal pathway. Cerebroprotection in the reperfusion era should target the neurovascular unit: delaying penumbral collapse before recanalization, limiting oxidative and vascular injury during thrombectomy, and restoring microcirculatory and barrier function afterwards. Recent trials show a differentiated landscape. ESCAPE-NEXT and RODIN were neutral; TASTE-2 suggested a borderline overall effect and imaging-defined heterogeneity for edaravone dexborneol; BAST supported potential benefit from butylphthalide; and the positive EMPHASIS trial contrasted with neutral EVT-specific MIST-A findings for minocycline. We organize mechanisms and interventions along the macrovascular recanalization–tissue reperfusion–neurovascular-unit preservation–functional recovery continuum, stratify evidence across pharmacological, physiological, delivery, natural-product, and cell/extracellular-vesicle strategies, and propose a precision framework integrating tissue and target windows, reperfusion status, and measurable treatment response. Next-generation trials should combine target engagement, imaging enrichment, prespecified biomarker interactions, and adaptive designs. Current evidence supports redesigning cerebroprotection, not adopting any candidate as a universal adjunct to reperfusion.

## 1. Introduction: The Persistent Gap Between Recanalization, Reperfusion, and Recovery

Mechanical thrombectomy has reshaped the natural history of stroke due to large-vessel occlusion. The HERMES collaboration and related trials established the substantial efficacy of endovascular therapy and expanded the therapeutic window beyond conventional time-based criteria to an imaging-defined tissue window [1,2]. However, although contemporary thrombectomy achieves successful angiographic reperfusion, commonly defined as modified Thrombolysis in Cerebral Infarction (mTICI) grade 2b–3, in most patients, approximately half of those with successful recanalization remain functionally dependent or have died by 90 days, a phenomenon commonly referred to as futile recanalization [3]. This paradox cannot be explained simply by delayed treatment. Advanced age, baseline stroke severity, ischemic core volume, collateral status, glycemia, systemic comorbidities, white matter injury, anesthesia, and peri-procedural hemodynamics all shape clinical outcomes. Moreover, microcirculatory failure, blood–brain barrier disruption, and secondary cell death after macrovascular reopening may further deplete otherwise salvageable tissue [1,2,3].

Accordingly, the central clinical challenge in the reperfusion era has shifted from whether the occluded artery can be reopened to how arterial recanalization can be translated into tissue salvage. Three related levels should be distinguished. Recanalization refers to the reopening of the occluded arterial lumen; tissue reperfusion denotes effective restoration of blood flow within the capillary bed and ischemic tissue; and functional outcome reflects tissue preservation together with network reorganization, compensatory recruitment of distributed brain regions, rehabilitation, and patient-level factors. Neurovascular-unit preservation may support these processes but is not an absolute prerequisite for improvement [4,5,6]. These levels are closely linked but not equivalent. The goal of cerebroprotection is therefore not to replace reperfusion but to delay penumbral collapse before reperfusion, limit oxidative and vascular injury during recanalization and early reperfusion, mitigate no-reflow, edema, and inflammation after reperfusion, and create a permissive milieu for subacute repair.

## 2. Literature Search Strategy and Evidence Selection

This article is a narrative review. Searches were conducted in PubMed and ClinicalTrials.gov from database inception through 4 July 2026. No lower publication-date limit or language restriction was applied. Searches were performed across all searchable fields and were not restricted to titles or abstracts. The terms below are reported as a descriptive concept set rather than as a verbatim database query. This concept set combined terms relating to acute ischemic stroke and reperfusion (acute ischemic stroke, thrombectomy, thrombolysis, recanalization, and reperfusion) with concepts relating to cerebroprotection and the neurovascular unit (neuroprotection, cerebroprotection, brain cytoprotection, neurovascular unit, no-reflow, blood–brain barrier, oxidative stress, inflammation, edema, and hemorrhagic transformation). The named agents, devices, biomarkers, and trials discussed below were assessed within this topic-based narrative evidence synthesis. Four authors independently screened the retrieved records for relevance. Disagreements regarding study selection were resolved through discussion among all four screeners until consensus was reached. We prioritized randomized controlled trials, prespecified subgroup analyses, systematic reviews, consensus statements, and human mechanistic studies; preclinical literature was included when it directly informed mechanisms, delivery, reproducibility, or translational readiness. Evidence selection was guided by relevance to the questions addressed in this Review. Because this was a narrative rather than systematic review, no formal meta-analysis or pooled effect estimation was performed. The revised reporting structure was informed by the Scale for the Assessment of Narrative Review Articles (SANRA) [7]; no formal item-by-item SANRA score was assigned.

## 3. From Neuroprotection to Brain Cytoprotection: A Neurovascular Unit Perspective

Traditional neuroprotection has largely focused on neuronal survival. Stroke Treatment Academic Industry Roundtable (STAIR) X and XI recommend the terms “brain cytoprotection” or “cerebroprotection” when the therapeutic goal is to protect the brain as an integrated tissue system, because stroke injury encompasses not only neurons but also astrocytes, oligodendrocytes, microglia, endothelial cells, pericytes, the basement membrane, white matter, and circulating immune cells [8,9]. The neurovascular unit is not a mere static anatomical aggregate; rather, it is a dynamic system organized through neurovascular coupling, metabolic substrate transport, ionic homeostasis, barrier function, and immune signaling [10,11,12,13]. During focal ischemia, different cell types exhibit distinct thresholds of vulnerability. Consequently, the effects of a cell-targeted intervention may vary by treatment phase and may extend beyond neuronal survival to astrocytic support, angiogenesis, and immune-mediated debris clearance [10,11,12,13].

**Figure 1:**
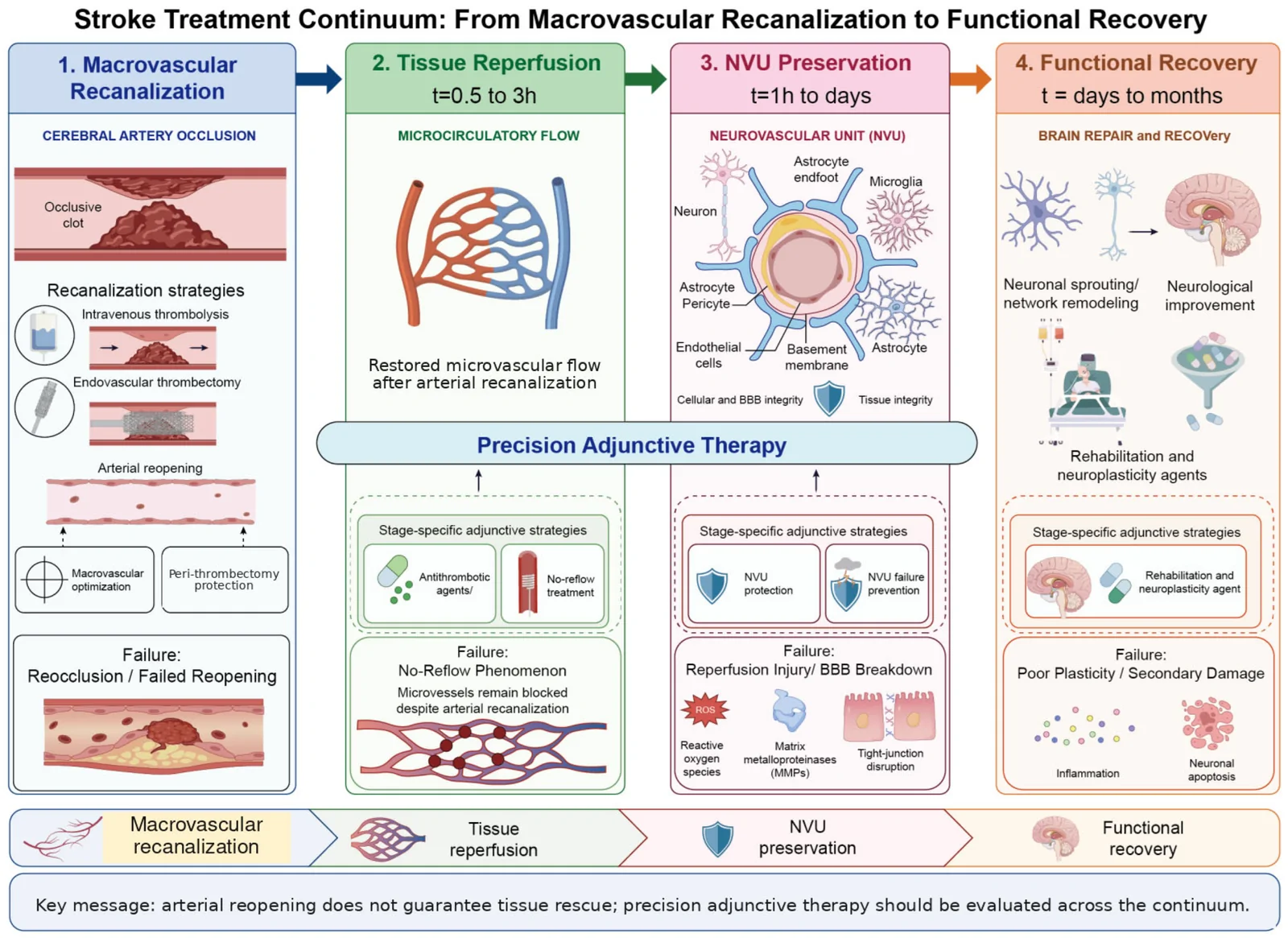
Continuum from macrovascular recanalization to functional recovery. Macrovascular reopening is the first step in the therapeutic continuum; microvascular no-reflow, reperfusion injury, and subsequent limitations in network recovery can progressively erode treatment benefit. Candidate precision adjunctive therapies may be evaluated at distinct stages—before recanalization, during the peri-thrombectomy period, after recanalization, and in the subacute phase—according to the tissue window, target window, reperfusion status, and measurable treatment response. The continuum is conceptual and does not imply that neurovascular-unit preservation is an absolute prerequisite for all functional improvement. Abbreviations: BBB, blood–brain barrier; NVU, neurovascular unit. Artwork statement: Figure 1 is an original illustration created by the authors using Adobe Illustrator 2024. Scientific-content statement: The conceptual content represents the authors’ synthesis of the literature cited in Section 1, Section 2 and Section 3 [1,2,3,4,5,6,7,8,9,10,11,12,13,14,15,16,17] and was not adapted or reproduced from any single published figure.

This perspective gives rise to two clinically relevant translational concepts. The first is the tissue window, which asks whether salvageable tissue remains in a given patient. The second is the target window, which defines when a given pathological process is a dominant driver of injury and can be safely modulated [9]. The target window for excitotoxicity and membrane depolarization may be measured in minutes to hours; oxidative stress extends across the pre- and post-recanalization periods; endothelial permeability and edema evolve over hours to days; and immune responses gradually shift from early amplification of injury to debris clearance and repair. Failure to account for the target window is an important explanation for many interventions that are mechanistically sound yet clinically ineffective. The conceptual continuum from arterial recanalization to tissue reperfusion and functional recovery is illustrated in Figure 1.

## 4. Why Traditional Neuroprotection Has Failed

### 4.1. Mismatch Between Preclinical Models and Clinical Stroke

Many candidate agents were derived from transient middle cerebral artery occlusion models in young, healthy, male rodents, whereas patients with stroke in clinical practice are typically older and frequently have comorbid hypertension, diabetes, atrial fibrillation, atherosclerosis, chronic kidney disease, and cerebral small vessel disease. Anesthesia, temperature control, occlusion duration, and highly standardized reperfusion in animal experiments differ substantially from the heterogeneity encountered in clinical stroke. Historical reviews have shown that thousands of experimental interventions yielded positive results in animals, yet few underwent rigorous validation across multiple centers, species, and comorbidity models [14,15,16,17]. Inadequate randomization, blinding, sample-size calculation, reporting of excluded animals, and publication of negative findings have also contributed to systematic inflation of effect sizes.

### 4.2. Mismatches in Treatment Timing, Brain Exposure, and Dose Selection

A major translational mismatch is that many laboratory neuroprotection studies initiate treatment before ischemia or within minutes of arterial occlusion, often before or at model reperfusion, whereas clinical administration usually follows emergency transport, imaging, eligibility assessment, and reperfusion workflow and may occur several hours after onset [14,15,16,17]. This shift can miss the period of peak target activity. Other studies failed to demonstrate adequately that candidate agents crossed the blood–brain barrier, achieved effective unbound concentrations, or produced target engagement. Blood–brain barrier permeability after stroke does not increase uniformly, and drug delivery to the severely ischemic core may be reduced because perfusion is absent. Before future candidates advance to pivotal trials, dose–exposure–response relationships, target engagement in brain tissue or validated surrogate compartments, interactions with alteplase or tenecteplase, and pharmacokinetic profiles under conditions of successful and unsuccessful reperfusion should be established [14,15,16,17].

### 4.3. Single-Target Agents Cannot Adequately Address Parallel Injury Networks

The ischemic cascade is not a linear pathway. Ionic imbalance, oxidative stress, mitochondrial failure, inflammation, thromboinflammation, and vascular permeability form self-reinforcing feedback loops [12,13]. Blockade of a single receptor may be bypassed by compensatory pathways, whereas excessive suppression of physiological signaling may cause toxicity. A multi-target strategy should therefore not be equated with “the more mechanisms, the better.” Rather, it should involve coordinated modulation of interconnected pathological nodes within the same disease endotype and should be accompanied by measurable pharmacodynamic biomarkers.

### 4.4. Failure to Align Trial Design with the Reperfusion Workflow

Most previous studies evaluated candidate agents as stand-alone therapies; only a minority of patients received thrombolysis, and almost none were treated in the context of contemporary stent-retriever endovascular thrombectomy (EVT). In the reperfusion era, trials should specify whether an agent is intended as a bridging therapy, a peri-thrombectomy intervention, or a post-reperfusion treatment. Concomitant thrombolytic therapy can alter candidate-drug exposure or interaction, as illustrated by the possible degradation of nerinetide by alteplase in ESCAPE-NA1 [18]. Thrombectomy also permits collection of arterial blood, venous blood, and thrombus samples for translational analyses [19,20] and may create opportunities for local intra-arterial delivery. However, ESCAPE-NEXT, which excluded thrombolysis, still failed to demonstrate efficacy, indicating that a single drug–drug interaction cannot fully explain therapeutic failure [21].

### 4.5. Inadequate Patient Selection and Endpoint Design

Broad eligibility criteria based only on time from onset and National Institutes of Health Stroke Scale (NIHSS) score may pool patients with completed infarction, substantial residual penumbra, severe blood–brain barrier disruption, or predominantly microcirculatory dysfunction, thereby diluting treatment effects confined to specific endotypes. Exclusive reliance on a dichotomized 90-day modified Rankin Scale (mRS) outcome may also miss mechanistic effects on infarct growth, edema, hemorrhage, cognition, and quality of life. Conversely, reliance on imaging surrogate endpoints alone cannot establish patient-centered benefit. Robust trial design should therefore pair functional outcomes with prespecified mechanistic endpoints while appropriately controlling for multiple comparisons [8,9].

## 5. Neurovascular Unit Injury Mechanisms and Therapeutic Targets in the Reperfusion Era

### 5.1. Excitotoxicity, Ionic Imbalance, and Spreading Depolarization

Reduced cerebral blood flow leads to adenosine triphosphate (ATP) depletion, Na^+^/K^+^-ATPase failure, membrane depolarization, and excessive glutamate release. N-methyl-D-aspartate (NMDA) receptors and voltage-gated calcium channels mediate Ca^2+^ overload, thereby activating proteases, phospholipases, nitric oxide synthase, and mitochondrial permeability transition [12,13]. This process is particularly relevant during the early phase of the ischemic penumbra and provides the rationale for long-standing interest in NMDA antagonists. However, nonselective NMDA receptor blockade interferes with normal synaptic function and can cause neuropsychiatric adverse effects. More selective strategies aim to modulate pathological receptor complexes, downstream postsynaptic density protein 95 (PSD-95) signaling, or GluN2B (NR2B)-associated toxicity while preserving physiological neurotransmission. Nerinetide and nelonemdaz represent downstream protein–protein interaction inhibition and NR2B-selective/free-radical-scavenging approaches, respectively; their negative confirmatory findings indicate that treatment timing, tissue endotype, and adequacy of target coverage require renewed scrutiny [18,21,22,23].

### 5.2. Oxidative Stress and Mitochondrial Injury

Reperfusion restores oxygen and substrate supply; however, damaged mitochondria, xanthine oxidase, nicotinamide adenine dinucleotide phosphate (NADPH) oxidase, and inflammatory cells can trigger a surge in reactive oxygen species (ROS). ROS injure lipids, proteins, and DNA, induce loss of mitochondrial membrane potential, cytochrome c release, and bioenergetic failure, and are mechanistically linked to matrix metalloproteinase (MMP) activation, increased endothelial permeability, and ferroptosis [12,13]. The clinical translation of antioxidant strategies has been challenging, partly because ROS biology depends on subcellular localization and timing, and partly because systemic scavengers may fail to reach ischemic tissue during the peak injury phase. Edaravone dexborneol, uric acid, and nelonemdaz exemplify different antioxidant paradigms, whereas mitochondria-targeted or ROS-responsive delivery systems may improve spatial selectivity [23,24,25,26].

### 5.3. Blood–Brain Barrier Disruption, Hemorrhagic Transformation, and Cerebral Edema

The blood–brain barrier is maintained by endothelial tight junctions, pericytes, the basement membrane, and astrocytic endfeet. Ischemia and reperfusion induce endothelial cytoskeletal remodeling, tight-junction protein degradation, basement-membrane disruption, and increased transcellular transport; barrier responses are also heterogeneous along the arteriovenous axis [27,28,29,30]. Matrix metalloproteinase-9 (MMP-9), inflammatory mediators, ROS, and the coagulation–fibrinolysis system collectively drive vasogenic edema and hemorrhagic transformation. A meta-analysis of 30 studies including 2609 patients showed that computed tomography (CT)- and magnetic resonance (MR)-based permeability indices were associated with hemorrhagic transformation, although effect estimates varied substantially and publication bias was evident [31]. Studies of the hyperintense acute reperfusion marker (HARM) and recent EVT cohorts further support the value of contrast extravasation or permeability abnormalities for risk stratification [32,33,34]. However, these measures currently function mainly as prognostic or safety biomarkers and have not been shown to select patients for a specific barrier-protective therapy. Severe edema may also compress capillaries by increasing tissue pressure, thereby worsening no-reflow. Upregulation of the sulfonylurea receptor 1–transient receptor potential melastatin 4 (SUR1-TRPM4) channel after ischemia provides a rationale for glibenclamide as an edema-targeted candidate therapy, but the neutral primary efficacy result of CHARM indicates that more precise enrichment for edema risk and more rigorous definition of ischemic core volume are needed [35].

### 5.4. Neuroinflammation, Thromboinflammation, and Immune Cell Infiltration

Ischemia-injured cells release damage-associated molecular patterns (DAMPs), thereby activating Toll-like receptor 4 (TLR4) signaling, inflammasomes, and complement pathways. Microglia, endothelial cells, and astrocytes produce chemokines that recruit neutrophils, monocytes, and lymphocytes [36]. Neutrophils can form neutrophil extracellular traps (NETs), obstruct capillaries, and increase thrombus resistance to lysis, whereas platelet–leukocyte aggregation and endothelial adhesion further amplify microcirculatory dysfunction. Thrombectomy-derived human specimens provide a critical translational bridge: Laridan et al. detected NETs in nearly all of 68 retrieved thrombi, and prospective cohort studies also identified associations of NET markers with histological composition and clinical outcomes in peripheral blood and 106 retrieved thrombi [37,38]. Integrated evidence from human brain tissue, plasma, and animal experiments further supports NET-related microvascular thrombosis, although therapeutic evidence remains confined to animal models [39]. Thus, biological relevance in humans cannot substitute for randomized validation of anti-NET interventions. Inflammation is also not uniformly deleterious: debris clearance, angiogenesis, and tissue remodeling during the subacute phase depend on selected immune signals. ApTOLL, minocycline, and fingolimod target TLR4, microglia/MMP-related inflammation, and lymphocyte migration, respectively, reflecting a shift from broad immunosuppression toward temporally informed mechanistic modulation [36,40,41].

### 5.5. Ferroptosis, Pyroptosis, and Necroptosis: Intersecting Networks of Regulated Cell Death

Ferroptosis is driven by iron-dependent lipid peroxidation, glutathione depletion, and failure of glutathione peroxidase 4 (GPX4)-mediated antioxidant defense. Pyroptosis is commonly linked to the NOD-like receptor protein 3 (NLRP3)–caspase-1–gasdermin axis, whereas necroptosis involves receptor-interacting protein kinases 1 and 3 (RIPK1/RIPK3) and mixed-lineage kinase domain-like protein (MLKL) signaling. Animal studies have linked disrupted iron homeostasis to post-stroke ferroptosis and suggest that iron accumulation during early reperfusion may promote both ferroptosis and necroptosis [42,43]. These regulated cell-death pathways interact with mitochondrial ROS, lysosomal injury, inflammation, and blood–brain barrier disruption, rather than representing mutually exclusive terminal programs. Human evidence remains limited. One study integrating data from 51 patients with stroke, 69 controls, brain organoids, and mouse experiments linked elevated serum hypoxanthine to gasdermin E (GSDME)-related endothelial pyroptosis, providing rare cross-level evidence but not clinical therapeutic validation [44]. Evidence for ferroptosis and necroptosis in humans is derived mainly from transcriptomic profiles, metabolites, or nonspecific protein biomarkers. Therefore, these pathways are useful for defining endotypes and pharmacodynamic endpoints, but they should not be portrayed as already having established clinical actionability.

### 5.6. Microcirculatory Dysfunction and No-Reflow

**Figure 2:**
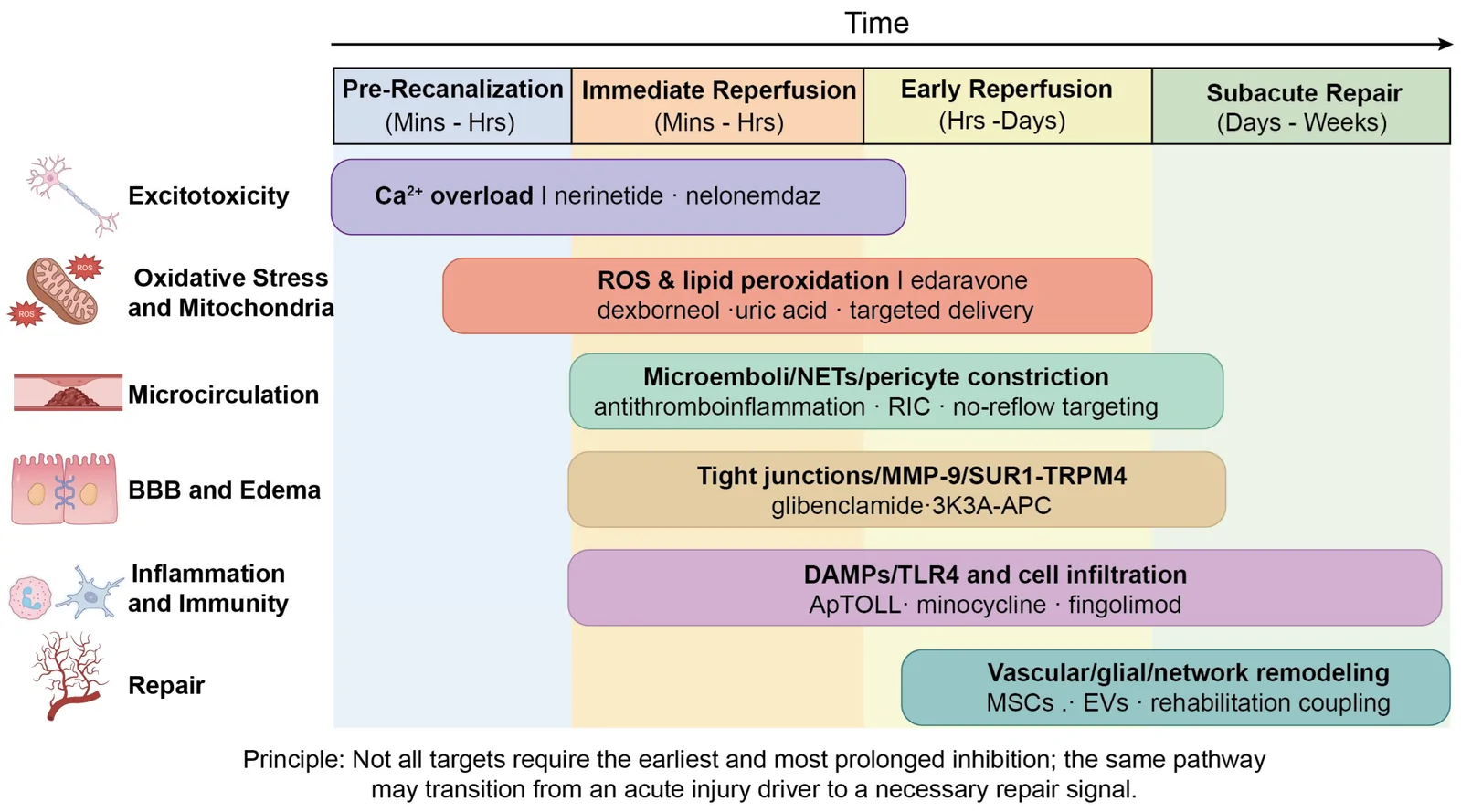
Neurovascular-unit pathology and candidate intervention windows across the ischemia–reperfusion timeline. Excitotoxicity and Ca^2+^ overload arise earliest; oxidative and mitochondrial injury span the pre- and post-recanalization periods; microemboli, neutrophil extracellular traps, pericyte constriction, blood–brain barrier disruption, edema, and inflammatory-cell infiltration evolve over hours to days; and vascular, glial, and network remodeling become increasingly relevant during subacute repair. Named agents or strategies are investigational or trial-tested examples linked to these processes; they are not established stage-specific standards. The bands represent overlapping conceptual windows of biological activity rather than validated clinical treatment boundaries. Abbreviations: 3K3A-APC, 3K3A-activated protein C; BBB, blood–brain barrier; Ca^2+^, calcium ion; DAMPs, damage-associated molecular patterns; EVs, extracellular vesicles; MMP-9, matrix metalloproteinase-9; MSCs, mesenchymal stromal cells; NETs, neutrophil extracellular traps; RIC, remote ischemic conditioning; ROS, reactive oxygen species; SUR1-TRPM4, sulfonylurea receptor 1–transient receptor potential melastatin 4; TLR4, Toll-like receptor 4. Artwork statement: Figure 2 is an original illustration created by the authors using Adobe Illustrator. Scientific-content statement: The conceptual content represents the authors’ synthesis of the literature cited in Section 3, Section 4, Section 5 and Section 6 [8,9,10,11,12,13,22,23,24,25,26,27,28,29,30,31,32,33,34,35,36,37,38,39,40,41,42,43,44,45,46,47,48,49,50,51,52,53,54,55,56,57,58,59,60,61,62] and was not adapted or reproduced from any single published figure.

No-reflow refers to persistent hypoperfusion within the downstream microvascular bed despite successful large-vessel recanalization. Its mechanisms include distal microembolization, erythrocyte and platelet aggregation, neutrophil plugging, endothelial swelling, sustained pericyte contraction, swelling of the basement membrane and astrocytic endfeet, and increased interstitial pressure [45,46,47,48]. Two-photon imaging has demonstrated pericyte contraction, neutrophil-mediated capillary obstruction, and persistent capillary dysfunction after reperfusion [49,50,51], supporting the microcirculation as a distinct therapeutic target layer. Clinically, a meta-analysis of 13 studies including 719 patients estimated the pooled incidence of no-reflow at 29% (95% confidence interval, 21–37%) and found it to be associated with a significant reduction in functional independence, although definitions varied substantially across studies [52]. Arterial spin-labeling magnetic resonance imaging (ASL-MRI), angiography, and immediate post-thrombectomy computed tomography (CT) perfusion have all been used to identify tissue reperfusion failure [53,54,55]. In a cohort of 151 patients, a smaller hypoperfusion volume on post-thrombectomy CT perfusion was associated with better distal reperfusion and functional outcomes, although this remains an observational association [55]. A 2025 post hoc pooled analysis of randomized trial data further showed that patients with complete angiographic recanalization but persistent tissue hypoperfusion had outcomes approaching those of patients in whom thrombectomy failed [56]. No-reflow should not be used interchangeably with futile recanalization: the former is a microcirculatory physiological phenotype, whereas the latter is a clinical definition of poor outcome despite successful recanalization. Standardization of imaging timing, modality, and thresholds is a prerequisite for introducing microcirculation-targeted therapies into clinical trials. Figure 2 summarizes the overlapping conceptual windows of neurovascular-unit injury and candidate interventions.

## 6. Pharmacological Cerebroprotection as an Adjunct to Reperfusion Therapy

### 6.1. Nerinetide: Mechanistic Plausibility but Negative Confirmatory Trials

Nerinetide was designed to inhibit pathological excitotoxicity by disrupting the NMDA receptor–PSD-95 complex while preserving physiological receptor function. In ESCAPE-NA1, nerinetide did not improve the primary functional endpoint in the overall population, although an exploratory signal of benefit was observed among patients who did not receive alteplase [18]. ESCAPE-NEXT subsequently enrolled patients with anterior-circulation large-vessel occlusion who had not received intravenous fibrinolysis and were intended for EVT. Among 850 patients, 90-day mRS 0–2 occurred in 45% of the nerinetide group and 46% of the placebo group, with an adjusted odds ratio (OR) of 0.97, and efficacy was not demonstrated [21]. The prehospital FRONTIER trial likewise failed to meet its prespecified primary endpoint [57]. Thus, nerinetide should be framed as a rigorous example of failed precision redesign rather than as a therapy approaching clinical success. Early-window individual-patient-data analyses may remain useful for hypothesis generation, but they cannot substitute for confirmatory trial evidence.

### 6.2. Nelonemdaz: Multi-Target Activity Does Not Automatically Translate into Clinical Benefit

Nelonemdaz combines NR2B-selective antagonism with free-radical scavenging activity. The phase II SONIC study did not show a significant difference in the proportion of patients achieving mRS 0–2, although a favorable directional signal was observed and the safety profile was acceptable [22]. The phase III RODIN trial enrolled 496 patients planned to undergo EVT within 12 h across 24 centers in South Korea. The trial showed no difference in the 12-week mRS shift analysis, with a common OR of 0.95; nor were infarct volume or symptomatic intracranial hemorrhage significantly reduced [23]. These findings indicate that even when a candidate agent targets both excitotoxicity and oxidative stress, mismatches in treatment initiation, effective exposure, reperfusion quality, or patient endotype may still preclude an overall functional benefit.

### 6.3. Edaravone and Edaravone Dexborneol: From Broad-Spectrum Mechanisms to Imaging Enrichment

Edaravone has free-radical scavenging activity, but its international evidence base is constrained by geographically concentrated use, study size, and comparator design. Edaravone dexborneol combines edaravone with dexborneol and is designed to modulate both oxidative stress and inflammation. In TASTE-SL, a sublingual formulation increased the proportion of patients achieving 90-day mRS 0–1 from 54.7% to 64.4% among 914 patients [24], although the trial was not specifically designed for a contemporary EVT population. More directly relevant to this Review, TASTE-2 enrolled 1362 patients with anterior-circulation large-vessel occlusion who were intended to undergo EVT; the study drug was initiated before thrombectomy and continued for 10–14 days. Functional independence at 90 days occurred in 55.0% versus 49.6% of patients, respectively, yielding a risk ratio (RR) of 1.11 (95% confidence interval, 1.00–1.23; *p* = 0.05) [25]. The overall effect was borderline. Although the prespecified mismatch subgroup showed a larger effect with a significant interaction, this finding requires dedicated validation. The main implication of TASTE-2 is not that it definitively proved drug efficacy, but that imaging-defined salvageable tissue may determine the treatment effect of multi-target cerebroprotection.

### 6.4. Butylphthalide: Positive Randomized Evidence in a Reperfusion-Treated Population

DL-3-n-butylphthalide has pleiotropic effects on mitochondrial function, microcirculation, oxidative stress, and inflammation. The BAST trial enrolled 1216 patients from 59 centers in China who received intravenous thrombolysis, EVT, or bridging therapy within 6 h. Using a baseline severity–adjusted sliding dichotomous definition of favorable outcome, the rate of favorable outcome was 56.7% versus 44.0%, with an OR of 1.70; serious adverse events were similar between groups [58]. This trial provides relatively strong positive evidence in a reperfusion-treated population. However, interpretation should still consider the single-country study population, combined treatment regimen, sliding dichotomous primary endpoint, and limited external replication. 

### 6.5. Minocycline: Contrasting Overall Benefit with EVT-Specific Neutral Findings

Minocycline modulates microglial activation, MMP activity, apoptosis, and inflammation. The 2026 EMPHASIS trial randomized 1724 patients with ischemic stroke within 72 h of onset across 58 hospitals in China. Short-course oral minocycline increased the proportion of patients achieving a 90-day mRS score of 0–1 from 47.4% to 52.6%, with an adjusted RR of 1.11 (95% CI, 1.03–1.20; *p* = 0.0061), and overall safety was similar between groups [59]. By contrast, the contemporaneous MIST-A trial randomized 189 patients with successful EVT recanalization across 8 hospitals and did not support a functional benefit of minocycline in this population [60]. Differences in enrollment severity, treatment window, reperfusion status, and pathological endotype mean that the two studies should not be viewed as directly contradictory. Nevertheless, they clearly show that overall efficacy in ischemic stroke does not necessarily imply specific adjunctive efficacy after successful recanalization. EMPHASIS requires cross-regional replication, whereas MIST-A indicates that peri-thrombectomy therapies must demonstrate direct engagement of microcirculatory, BBB-related, or other post-reperfusion mechanisms.

### 6.6. ApTOLL, 3K3A-Activated Protein C, and Immune–Vascular Protection

ApTOLL is a deoxyribonucleic acid (DNA) aptamer that binds TLR4. In the APRIL phase I/II study, pre-EVT administration at 0.2 mg/kg was associated with lower 90-day mortality, smaller infarct volume, and a more favorable mRS distribution. However, because the primary objective was safety, the sample size was small, and interpretation is complicated by multiple dose comparisons, these efficacy signals require confirmation in pivotal trials [40,61]. 3K3A-activated protein C (3K3A-APC) is an activated protein C variant engineered to reduce anticoagulant activity while preserving cytoprotective signaling. RHAPSODY showed an acceptable safety profile when 3K3A-APC was combined with thrombolysis and/or thrombectomy, with an exploratory signal toward reduced bleeding burden [62]. Together, these agents illustrate a shift from neuron-centered receptor targeting toward immune–endothelial–barrier protection.

### 6.7. Uric Acid, Magnesium, and the Lesson That Earlier Treatment Is Necessary but Insufficient

URICO-ICTUS did not demonstrate an improvement in 90-day functional outcomes with uric acid in the overall population, and signals in subgroups such as women or patients with hyperglycemia should be regarded only as hypothesis-generating [26]. FAST-MAG successfully demonstrated the feasibility of ultra-early prehospital randomization and treatment administration, but the trial, which enrolled more than 1700 patients, did not show an improvement in the primary functional outcome with magnesium [63]. Together, these studies indicate that earlier treatment is one necessary condition, but not a sufficient one. Candidate agents must still target the relevant pathological drivers and achieve a sufficient effect size in patients with salvageable tissue.

### 6.8. Glibenclamide, Cerebral Edema, and Large-Core Stroke

Intravenous glibenclamide targets cytotoxic and vasogenic edema through inhibition of the SUR1-TRPM4 channel. GAMES-RP did not improve the primary functional endpoint, but it reduced edema-related measures, including midline shift and MMP-9 [64]. The phase III CHARM trial randomized 535 patients, of whom 431 patients aged 18–70 years comprised the prespecified modified intention-to-treat efficacy population. Intravenous glibenclamide did not improve the primary 90-day ordinal mRS outcome in this population (common OR, 1.17; 95% CI, 0.80–1.71; *p* = 0.42) [35]. Subsequent analyses stratified by core volume and EVT status suggested potential effects, but the confidence intervals were wide, and these findings should be considered hypothesis-generating [35]. This program illustrates that positive mechanistic endpoints and a negative functional endpoint in a prespecified efficacy population are not necessarily contradictory. The critical issue is enrichment for patients in whom edema is a major determinant of prognosis.

### 6.9. Fingolimod: The Need for Confirmation After Small Studies

Fingolimod modulates sphingosine-1-phosphate receptors, thereby reducing peripheral lymphocyte circulation and lymphocyte infiltration into the brain. A trial of approximately 47 patients receiving fingolimod in combination with alteplase showed smaller lesion volumes, reduced hemorrhage, and better functional outcomes, and a study in the delayed thrombolysis window also suggested improved antegrade perfusion and collateral blood flow [41,65]. However, the small sample size, open-label design, and immune and cardiac safety concerns limit the strength of inference. Until multicenter blinded validation is available, fingolimod should not be presented on the same evidentiary footing as large phase III evidence. Table 1 summarizes selected clinical trials discussed in Section 6 and Section 7.

## 7. Non-Pharmacological and Physiological Cerebroprotective Strategies

### 7.1. Remote Ischemic Conditioning

Remote ischemic conditioning (RIC) induces humoral, neural, and immune protective signals through repeated brief cycles of limb ischemia and reperfusion. In RICAMIS, among 1893 patients with moderate stroke who did not receive reperfusion therapy, the proportion achieving 90-day mRS 0–1 was 67.4% versus 62.0%; however, the trial did not include a sham-control procedure, and treatment was continued for 10–14 days [69]. By contrast, RESIST, a prehospital-initiated, sham-controlled trial of 1500 patients with suspected stroke, did not improve the 90-day mRS shift among confirmed stroke cases [66]. RICAIS in 2026 also showed no benefit, although it enrolled only 79 patients [70]. The discrepant findings may reflect differences in study population, dose, treatment duration, reperfusion status, and blinding. At present, RIC appears safe and low-cost, but its efficacy remains unestablished; it is therefore best suited for re-evaluation in platform-style studies with rich mechanistic biomarker assessment.

### 7.2. Therapeutic Hypothermia

Therapeutic hypothermia can simultaneously reduce metabolic demand, excitotoxicity, reactive oxygen species generation, inflammation, and blood–brain barrier permeability, making it a prototypical multi-target strategy. Major barriers include shivering in awake patients, pneumonia, arrhythmias, the need for sedation, delayed cooling, and rebound injury during rewarming. Large programs such as EuroHYP-1 encountered recruitment and feasibility barriers, and previous studies have not established evidence supporting routine use [67,71]. Selective brain cooling, thrombectomy catheter-based local cooling, and milder temperature management may improve the benefit–risk ratio, but these approaches must demonstrate actual brain-temperature reduction, target engagement, and no procedural delay.

### 7.3. Normobaric Hyperoxia

Normobaric hyperoxia aims to support penumbral metabolism before arterial recanalization by increasing dissolved oxygen in plasma. Small trials combining normobaric hyperoxia with EVT have suggested possible reductions in infarct volume or improvements in early neurological function [72]. The PROOF phase IIb protocol incorporated target-mismatch selection and brain-protective bridging until endovascular revascularization [73]. OPENS-2 randomized 282 patients and reported a favorable 90-day ordinal mRS shift (adjusted common OR, 1.65; 95% CI, 1.09–2.50; *p* = 0.018) without an observed increase in serious adverse events [68]. Its practical advantage is that it can be initiated rapidly after imaging and before EVT, whereas potential risks include hyperoxia-induced ROS generation and masking of hypoxemia. Future studies should treat oxygenation status, oxygen dose, timing of initiation and discontinuation, and timing relative to reperfusion as a complete therapeutic exposure, rather than merely recording whether oxygen was administered.

## 8. Nanomedicine and Targeted Delivery

Cerebroprotective candidates face three delivery paradoxes: the blood–brain barrier restricts access to the penumbra; the completely nonperfused ischemic core may receive little drug delivery even when the barrier is disrupted; and systemic administration may cause peripheral immune, coagulation-related, or organ toxicity. The value of nanocarriers is therefore not simply to increase total brain drug concentration, but to deliver therapeutic payloads, at the appropriate phase, to pathological microenvironments that remain perfused [74,75,76].

ROS-responsive nanoparticles: these systems exploit the oxidative microenvironment to trigger the release of antioxidants, mitochondrial protectants, or anti-inflammatory agents. Cell membrane–coated systems: erythrocyte membranes prolong circulation, whereas neutrophil or platelet membranes mimic inflammatory adhesion and thrombus targeting. BBB-penetrating carriers: these platforms mediate transendothelial transport through transferrin receptors, low-density lipoprotein receptor-related proteins, or cell-penetrating peptides. Thrombus/ischemic-microenvironment targeting: these strategies recognize activated platelets, fibrin, acidic pH, MMPs, or exposed endothelial adhesion molecules. Exosome-mimetic systems: these approaches preserve natural membrane compatibility while improving loading capacity, purity, and batch-to-batch consistency [74,75,76].

Recent systematic reviews have identified ischemic-microenvironment responsiveness, cell-membrane coating, receptor-mediated BBB transport, and thrombus targeting as major technical routes, but clinical evidence remains nearly absent [74]. A 2025 preclinical meta-analysis of nanoparticle-based thrombolytic agents included 18 in vivo studies and suggested reductions in infarct volume and improvements in neurological scores compared with free recombinant tissue plasminogen activator (rtPA); however, the analysis also showed substantial heterogeneity and funnel-plot asymmetry [75]. Effect sizes should therefore be interpreted in the context of risk of bias and the translational relevance of the underlying models. A 2026 review of delivery strategies still framed this field as a pre-translational platform rather than as a therapeutic category ready for integration into routine stroke workflows [76].

The vast majority of strategies remain at the rodent-model stage. Safety and manufacturability should be evaluated on an equal footing with efficacy: hepatic and splenic accumulation, complement activation, microthrombotic risk, long-term degradation, sterility and endotoxin control, manufacturing scale-up, freeze–thaw stability, and regulatory classification as a drug, device, or combination product may all impede translation [74,75,76]. In particular, increased brain fluorescence should not be taken as direct evidence of effective delivery across the BBB. Intravascular adhesion, endothelial uptake, and parenchymal release must be distinguished, and whole-body mass balance, pharmacokinetics, repeated-dose toxicology, and immunogenicity should be assessed. Minimum standards before pivotal validation should include dual controls with free drug and empty particles, aged and comorbid models, compatibility with intravenous thrombolysis (IVT) and EVT, scalable critical quality attributes, and potency assays linked to the mechanism of action. Good manufacturing practice (GMP) processes, batch-to-batch consistency, and release criteria should therefore be incorporated at the candidate-selection stage, together with pharmacokinetic/pharmacodynamic assessment [74,75,76].

## 9. Natural Product–Derived Therapies and Multi-Target Pharmacology

Natural products may provide chemical scaffolds with potential multi-target activity, but a multi-component composition does not necessarily translate into controllable multi-target pharmacology. Butylphthalide has evidence from a large randomized trial, which is discussed in Section 6.4 [58]. Puerarin and quercetin frequently demonstrate antioxidant, anti-inflammatory, mitochondrial, or BBB-protective effects in experimental studies, but their clinical translation has been constrained by low bioavailability, rapid metabolism, formulation variability, and variable study quality. A Cochrane review of puerarin included 20 trials and 1574 patients; because of incomplete methodological reporting, short follow-up, and insufficient data on death or dependency outcomes, the evidence remained insufficient to determine clinical efficacy [77]. Evidence for quercetin in stroke is also derived mainly from preclinical mechanistic studies [78]. These examples illustrate that elaborate pathway narratives cannot compensate for the lack of reliable patient-centered outcomes. This field should be held to the same standards as new molecular entities, including defined chemical composition, impurity profiling, batch-to-batch consistency, dose–exposure relationships, brain target engagement, and interactions with antithrombotic therapy. Network pharmacology and molecular docking can generate mechanistic hypotheses, but they cannot serve as evidence of efficacy or direct target engagement.

## 10. Cell- and Extracellular Vesicle–Based Therapies: From Acute Protection to Repair

Mesenchymal stromal cells (MSCs), umbilical cord–derived MSCs, neural stem cells, and multipotent adult progenitor cells may exert their effects through paracrine signaling, immunomodulation, angiogenesis, and neurotrophic support. They are better conceptualized as strategies for subacute immunomodulation and recovery enhancement rather than as hyperacute neuroprotective interventions operating on a minutes-scale. The phase II MASTERS trial did not meet its 90-day primary composite efficacy endpoint, although it demonstrated feasibility of administration [79]. TREASURE, which included 206 patients in Japan, also failed to meet its prespecified primary outcome, although some long-term or age-related analyses generated hypotheses [80]. Heterogeneity in cell source, culture conditions, potency, dose, route of administration, therapeutic window, and host immune status makes pooled findings from small trials difficult to translate directly into clinical recommendations.

MSC-derived extracellular vesicles (EVs) may transfer microRNAs (miRNAs), proteins, and lipids while avoiding some concerns associated with live cells, including embolism, survival, and tumor formation. Engineered EVs can be loaded with miRNAs or mitochondria-modulating molecules, but evidence for efficacy in stroke remains predominantly preclinical [81,82,83,84]. The Minimal Information for Studies of Extracellular Vesicles 2023 guideline (MISEV2023) requires clear reporting of the source, isolation methods, particle identity and non-vesicular contaminants, quantification, and functional characterization [85]. For drug development, standards for identity, purity, potency, stability, sterility and endotoxin control, residual host–cell components, tissue distribution, and safety release criteria must also be established [86,87]. Whether EVs are regulated as biological products, advanced therapy products, or combination products depends on their source, degree of engineering, and mechanism of action. Describing them as “natural nanomedicines” does not bypass the need for GMP manufacturing or definition of critical product attributes.

## 11. Precision Adjunctive Therapy: From Prognostic Stratification to Treatment Response Prediction

### 11.1. Imaging Endotypes

Imaging selection should not merely answer whether a patient should undergo thrombectomy; it can also help define endotypes relevant to cerebroprotection. Core–penumbra mismatch indicates the presence of salvageable tissue, whereas a low Alberta Stroke Program Early CT Score (ASPECTS) and a large ischemic core signal increased risks of edema and blood–brain barrier disruption. Collateral status, hypoperfusion intensity ratio (HIR), and venous outflow profiles reflect the pace of ischemic progression and microcirculatory reserve. Post-thrombectomy CT or magnetic resonance (MR) perfusion, digital subtraction angiography (DSA) tissue-staining patterns, and arterial spin labeling (ASL) can be used to identify no-reflow [52,53,54,55,88]. BBB permeability and iodine extravasation on dual-energy CT may further support hemorrhage-risk stratification [31,32,33,34]. A 2026 systematic review showed that these imaging markers, together with white matter disease and brain atrophy, were associated with futile recanalization; however, most studies were prognostic rather than predictive of treatment response [89]. A biomarker should be considered a true selection tool only when it shows a prespecified and reproducible interaction with randomized treatment effects.

### 11.2. Circulating and Local Biomarkers

Neurofilament light chain (NfL) reflects axonal injury, glial fibrillary acidic protein (GFAP) reflects astroglial and barrier-related injury, MMP-9 is associated with blood–brain barrier breakdown and hemorrhagic transformation, and inflammatory cytokines, the neutrophil-to-lymphocyte ratio (NLR), and complement markers reflect systemic–brain immune responses. However, prognostic association does not equate to treatment-selection utility. Prospective studies have shown that NfL and GFAP can complement outcome prediction, but they have not demonstrated an ability to identify patients likely to benefit from a specific cerebroprotective therapy [90,91,92]. Thrombectomy provides a unique sampling window: a proteomic study of 25 paired intracranial and peripheral blood samples and a transcriptomic–cytokine study of blood sampled distal and proximal to the occlusion in 28 patients both showed that the local ischemic environment differs from peripheral blood [19,20]. NfL measured distal to the occlusion was associated with outcome in a 110-patient cohort, but this remains prognostic evidence [93]. Local MMP-9 is also still at the proof-of-concept stage [94]. Biomarkers must therefore be validated separately for diagnostic, prognostic, predictive, pharmacodynamic, and safety purposes. Table 2 summarizes candidate biomarkers, their intended uses, and the current maturity of the evidence.

### 11.3. Multi-Omics and Single-Cell Technologies

Transcriptomic, proteomic, and metabolomic profiling of peripheral blood can identify inflammatory, oxidative, and coagulation-related endotypes. Retrieved thrombi, arterial blood, and postprocedural samples can link local immune status to reperfusion quality, whereas single-cell and spatial omics can resolve endothelial, pericyte, myeloid, and glial cell states. Recent mouse studies have generated spatial and single-cell transcriptomic atlases of the ischemic brain and revealed marked state divergence between brain-infiltrating leukocytes and their circulating counterparts [95,96]. These advances are useful for target discovery and for defining candidate endotypes, but cross-species mapping, sampling bias, and acute-phase feasibility in patients remain important limitations. Clinically meaningful multi-omics studies should incorporate prespecified sampling time points, independent validation cohorts, and measurable candidate panels, and should demonstrate biomarker-by-treatment interactions rather than merely improving outcome prediction.

### 11.4. Artificial Intelligence (AI): From Risk Prediction to Heterogeneity of Treatment Effect

**Figure 3:**
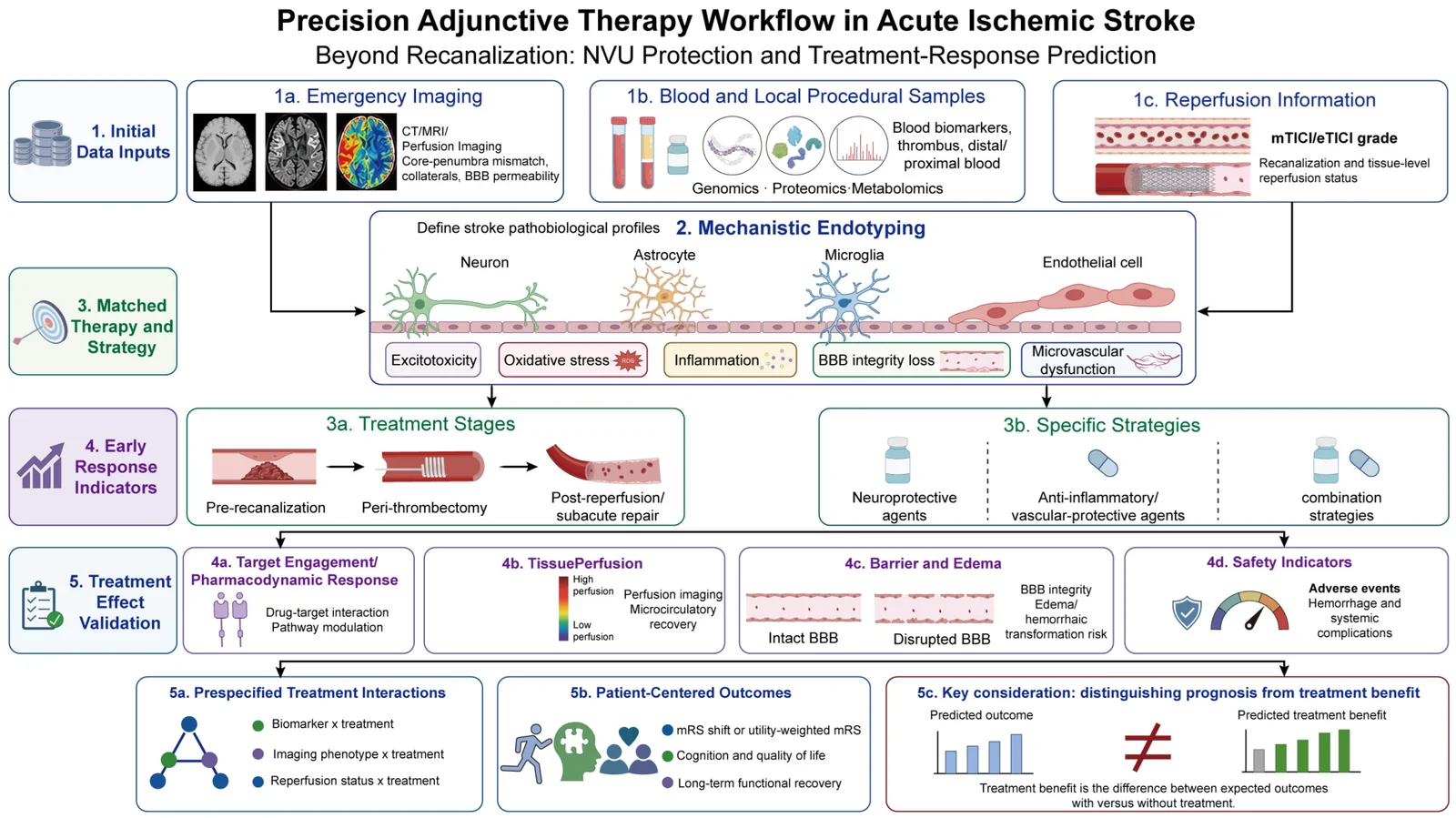
Precision adjunctive-therapy workflow. Emergency imaging, circulating and locally obtained samples, and reperfusion status are integrated to define mechanistic endotypes and guide selection of a candidate therapeutic phase and strategy. In the mechanistic-endotyping panel, the displayed cell–process pairings indicate predominant, nonexclusive associations: neurons with excitotoxicity and oxidative stress, microglia with inflammation, astrocytes with blood–brain barrier integrity loss, and endothelial cells with microvascular dysfunction. Target engagement, tissue perfusion, barrier integrity and edema, and safety serve as early response measures, whereas treatment effects are ultimately tested through prespecified treatment-by-biomarker interactions and patient-centered outcomes. Predicting prognosis is not equivalent to predicting treatment benefit. Abbreviations: BBB, blood–brain barrier; CT, computed tomography; eTICI, expanded Treatment in Cerebral Ischemia; MRI, magnetic resonance imaging; mRS, modified Rankin Scale; mTICI, modified Thrombolysis in Cerebral Infarction; NVU, neurovascular unit. Artwork statement: The schematic layout and non-imaging graphical elements in Figure 3 are original and were created by the authors using Adobe Illustrator. The three imaging thumbnails were generated using GPT-5.6 (OpenAI) on 10 July 2026 and are used solely for conceptual visualization; they do not depict real patients and were not derived from clinical imaging data. Scientific-content statement: The conceptual content represents the authors’ synthesis of the literature cited in Section 5 and Section 11 [31,32,33,34,52,53,54,55,56,88,89,90,91,92,93,94,95,96,97,98,99,100,101] and was not adapted or reproduced from any single published figure.

Most existing artificial intelligence (AI) studies predict futile recanalization, hemorrhage, or edema, and their outputs are largely prognostic probabilities. A scoping review covering 505 original studies showed that outcome prediction remains the dominant focus, whereas evidence for external validation and implementation is still limited [97]. Machine-learning studies of futile recanalization in stroke are likewise largely risk-prediction models [98]. Precision therapy, however, requires a counterfactual question to be answered: for the same patient, what difference in expected outcome would result from receiving versus not receiving a given cerebroprotective therapy? Causal machine-learning approaches, including causal forests, meta-learners, and Bayesian methods, can estimate heterogeneity of treatment effect, but they must guard against confounding, overfitting, and the erroneous assumption that high baseline risk necessarily implies greater treatment benefit [99,100,101]. Ideally, models should be trained and prespecified or frozen within randomized trials, followed by multicenter external validation, calibration, decision-curve analysis, and prospective silent-mode testing. Model inputs must also be available within the emergency stroke workflow. Figure 3 illustrates the authors’ proposed workflow for biomarker-informed adjunctive-therapy research.

## 12. Discussion: Evidence Synthesis and Next-Generation Trial Design

### 12.1. Interpretation of the Evidence

The literature reviewed above does not support a universal adjunctive agent; instead, it shows heterogeneous effects across timing, reperfusion status, tissue phenotype, and evidence maturity. STAIR X/XI and the 2026 Preclinical Ischemic Stroke Multicenter Trials (PRISM) statement emphasize compatibility with contemporary reperfusion workflows, reproducible preclinical evidence, target engagement, and interdisciplinary trial readiness [8,9,102,103]. The design principles below are therefore presented explicitly as the authors’ synthesis of the reviewed evidence, not as established clinical recommendations.

### 12.2. Authors’ Proposed Trial-Design Framework

Based on the evidence synthesized above, we propose the following framework for future trials:

Define strategies by treatment phase: prehospital or pre-recanalization bridging, peri-thrombectomy intervention, post-reperfusion treatment, and subacute repair should be evaluated separately.

Enrich patients by mechanism: for example, patients with salvageable penumbra may be enriched for excitotoxicity- or metabolism-targeted protection, those with BBB permeability abnormalities or large ischemic cores for edema-targeted protection, and those with persistent post-thrombectomy perfusion defects for microcirculatory therapies.

Prespecify stratification by intravenous thrombolytic type, degree of recanalization, first-pass effect, time, core volume, collateral status, and anesthetic strategy, and formally test treatment interactions.

Use a dual-tier endpoint structure: 90-day mRS shift or utility-weighted mRS should serve as patient-centered outcomes, while mechanism-aligned endpoints such as infarct growth, edema, BBB permeability, no-reflow, or target engagement should be assessed in parallel.

Extend follow-up to cognition, fatigue, depression, quality of life, and social participation at 6–12 months to avoid conflating motor recovery with global recovery.

Use adaptive platform or factorial designs to compare multiple candidates and their interactions with IVT/EVT efficiently, while rigorously controlling for multiplicity and temporal trends.

Complete multicenter preclinical validation, pharmacokinetic/pharmacodynamic assessment, brain-exposure studies, dose–response evaluation, and compatibility testing with contemporary reperfusion workflows before pivotal trials.

The mismatch interaction in TASTE-2, the large-core subgroup findings in CHARM, and the early-window hypothesis for nerinetide illustrate that subgroup findings can guide the next trial, but they cannot validate themselves within the same dataset [18,21,25,35]. The goal of next-generation studies should not be to search repeatedly for nominally positive subgroups within overall neutral trials but to validate reproducible treatment effects in independent, prespecified, and mechanistically interpretable populations.

### 12.3. Limitations of This Narrative Review

This narrative review does not provide exhaustive systematic screening, formal risk-of-bias assessment for every included study, or pooled effect estimation. The breadth of topics required selective rather than exhaustive coverage, and the rapidly evolving 2025–2026 trial landscape may alter specific conclusions. Several proposed delivery, biomarker, and cell-based strategies remain predominantly preclinical, and cross-study comparisons are limited by heterogeneity in populations, reperfusion workflows, timing, and endpoints. Finally, the stage-specific precision framework presented here is an author-proposed synthesis that requires prospective validation; it should not be interpreted as a clinical guideline. In addition, the search was limited to PubMed and ClinicalTrials.gov and was not designed as a prospectively registered, exhaustive multi-database systematic-review protocol.

## 13. Conclusions

Reperfusion therapy has largely addressed macrovascular reopening, but it has not fully resolved perfusion, barrier integrity, or cell survival at the tissue level [1,2,3,45,46,47,48,49,50,51,52,53,54,55,56]. Cerebroprotection has not become obsolete; rather, what is obsolete is the paradigm that reduces heterogeneous stroke to a single neuronal pathway, administers delayed single-target agents to broadly defined populations, and expects universal functional benefit. In our interpretation, cerebroprotection in the reperfusion era should target the neurovascular unit, be framed along the recanalization–tissue reperfusion–recovery continuum, use the tissue and target windows to guide therapeutic timing, and test treatment-response endotypes through imaging, biomarkers, and causal analysis [8,9].

As of 4 July 2026, our assessment of the evidence reviewed here identified no candidate cerebroprotective agent that had become an internationally accepted standard adjunct for all patients undergoing reperfusion. The confirmatory failures of nerinetide and nelonemdaz call for caution [21,23], whereas imaging-defined heterogeneity with edaravone dexborneol, results for butylphthalide in reperfusion-treated populations, divergence between EMPHASIS and MIST-A, and early signals for ApTOLL provide testable hypotheses for trial redesign [25,40,58,59,60,61]. Future progress is more likely to arise from testable combinations of reperfusion status, tissue windows, target windows, and treatment-response biomarkers than from assuming a single drug will benefit all patients with stroke.

## Figures and Tables

**Table 1:** Selected Clinical Trials of Neuroprotective Strategies in the Reperfusion Era.

Intervention/Trial	Proposed Target(s)	Design and Sample	Reperfusion Context	Primary Efficacy Finding	Evidence Interpretation
Nerinetide (ESCAPE-NEXT) [21]	NMDA receptor–PSD-95 uncoupling; excitotoxic signaling	Multicenter, double-blind, placebo-controlled confirmatory RCT; *n* = 850	Anterior-circulation LVO treated with EVT without preceding IVT	90-day mRS 0–2: 45% vs. 46%; OR 0.97 (95% CI 0.72–1.30); *p* = 0.82	Neutral confirmatory trial; no functional benefit in the prespecified no-thrombolysis population.
Nelonemdaz (RODIN) [23]	NR2B-selective NMDA antagonism; free-radical scavenging	Phase 3, multicenter, double-blind, placebo-controlled RCT; *n* = 496 randomized	Anterior-circulation LVO; mechanical reperfusion planned within 12 h	12-week ordinal mRS shift: cOR 0.95 (95% CI 0.69–1.31)	Neutral phase 3 trial; responder-subgroup analyses remain hypothesis-generating.
Edaravone dexborneol (TASTE-2) [25]	Oxidative stress and inflammatory injury	Multicenter, double-blind, placebo-controlled RCT; *n* = 1362 randomized; *n* = 1360 mITT	Anterior-circulation LVO within 24 h; study drug initiated before EVT	90-day mRS 0–2: 55.0% vs. 49.6%; RR 1.11 (95% CI 1.00–1.23); *p* = 0.05	Modest positive result with borderline precision; the mismatch-associated signal requires independent confirmation.
DL-3-n-butylphthalide (BAST) [58]	Mitochondrial function, microcirculation, oxidative stress, and inflammation	Multicenter, double-blind, placebo-controlled RCT; *n* = 1216	IVT and/or EVT; study treatment started within 6 h of symptom onset	Favorable 90-day outcome based on a sliding mRS dichotomy: 56.7% vs. 44.0%; OR 1.70 (95% CI 1.35–2.14); *p* < 0.001	Positive large RCT; geographic replication and regulatory confirmation remain important.
Minocycline (EMPHASIS) [59]	Neuroinflammation, microglial activation, and MMP-associated injury	Multicenter, double-blind, placebo-controlled RCT; *n* = 1724 randomized	Imaging-confirmed AIS treated within 72 h; not specific to EVT or IVT	90-day mRS 0–1: 52.6% vs. 47.4%; adjusted RR 1.11 (95% CI 1.03–1.20); *p* = 0.0061	Positive large RCT, but direct applicability to contemporary EVT-treated LVO is limited.
Minocycline (MIST-A) [60]	Neuroinflammation, microglial activation, and MMP-associated injury	Phase 2, multicenter, randomized, open-label, blinded-endpoint trial; *n* = 189 randomized; *n* = 187 mITT	Anterior-circulation LVO with successful reperfusion after mechanical thrombectomy	Day-5 infarct-growth ratio: 1.8 vs. 1.6; adjusted geometric mean ratio 1.10 (95% CI 0.84–1.45); *p* = 0.49. 90-day mRS 0–2: 68.1% vs. 72.0%; adjusted RR 1.00 (95% CI 0.70–1.43).	Neutral EVT-specific phase 2 trial; does not support adjunctive minocycline after successful thrombectomy.
ApTOLL (APRIL) [40]	TLR4-mediated innate immune activation	Phase 1b/2a, double-blind, placebo-controlled RCT; *n* = 151 randomized; selected-dose pooled cohort *n* = 139	Anterior-circulation LVO within 6 h; ApTOLL given before EVT, with IVT if indicated	Primary safety events with 0.2 mg/kg: 14% vs. 29% with placebo. Secondary 90-day ordinal mRS: cOR 2.44 (95% CI 1.76–5.00); final infarct volume difference −42% (95% CI −66% to 1%).	Promising safety and exploratory efficacy signals; not powered for definitive clinical efficacy and affected by baseline imbalances.
3K3A-APC (RHAPSODY) [62]	PAR1-mediated cytoprotection and blood–brain barrier stabilization	Multicenter, double-blind, placebo-controlled adaptive phase 2 dose-escalation trial; *n* = 110	IVT, mechanical thrombectomy, or both	Maximum tolerated dose: 540 μg/kg (estimated toxicity 7%). Exploratory pooled-dose analysis: any ICH 67.4% vs. 86.5% with placebo; *p* = 0.046.	Safety and tolerability established; hemorrhage findings were exploratory and require confirmatory efficacy testing.
Uric acid (URICO-ICTUS) [26]	Free-radical scavenging and antioxidant defense	Phase 2b/3, multicenter, double-blind, placebo-controlled RCT; *n* = 421 randomized; target population *n* = 411	Adjunct to alteplase within 4.5 h; only a small subgroup underwent thrombectomy	Excellent 90-day outcome: 39% vs. 33%; adjusted RR 1.23 (95% CI 0.96–1.56); *p* = 0.099	Neutral overall; thrombectomy and other subgroup findings are hypothesis-generating only.
Magnesium sulfate (FAST-MAG) [63]	Excitotoxicity and ionic homeostasis	Phase 3, prehospital, double-blind, placebo-controlled RCT; *n* = 1700 with suspected acute stroke	Treatment initiated within 2 h, before the modern EVT era; mixed ischemic and hemorrhagic stroke population	Mean 90-day mRS was 2.7 in both groups; no significant shift in disability (*p* = 0.28).	Neutral efficacy result; demonstrated the operational feasibility of ultra-early prehospital treatment.
Intravenous glibenclamide (CHARM) [35]	SUR1-TRPM4-mediated cerebral edema	Phase 3, double-blind, placebo-controlled RCT; *n* = 535 randomized; primary mITT population *n* = 431	Large hemispheric infarction; EVT permitted; trial stopped early for operational reasons	90-day ordinal mRS: cOR 1.17 (95% CI 0.80–1.71); *p* = 0.42. Mortality: 32% vs. 29%; HR 1.20 (95% CI 0.85–1.70).	Neutral and underpowered phase 3 trial; subgroup findings should not be considered confirmatory.
Fingolimod plus alteplase (pilot trial) [41]	S1P receptor signaling and lymphocyte trafficking	Three-center, open-label pilot RCT with coin-toss allocation; *n* = 47	Alteplase within 4.5 h; no EVT-specific design	Day-1 lesion growth: 10.1 vs. 34.3 mL (*p* = 0.04); hemorrhage volume: 1.2 vs. 4.4 mL (*p* = 0.01); 90-day mRS 0–1: 73% vs. 32% (*p* = 0.01).	Exploratory positive signal with substantial risk of bias and imprecision; requires a larger blinded trial.
Remote ischemic conditioning (RESIST) [66]	Humoral, neural, and immune conditioning pathways	Prehospital, randomized, patient- and assessor-blinded, sham-controlled trial; *n* = 1500 randomized; target stroke population *n* = 902	Suspected stroke within 4 h; reperfusion treatment provided according to standard care	90-day ordinal mRS in the target stroke population: OR 0.95 (95% CI 0.75–1.20); *p* = 0.67	Neutral sham-controlled trial; heterogeneous population and incomplete conditioning exposure may limit inference.
Endovascular hypothermia (ICTuS 2) [67]	Metabolic demand, excitotoxicity, oxidative stress, and inflammation	Randomized controlled phase 2 study; *n* = 120 of a planned 1600 before early termination	Cooling after intravenous alteplase; intra-arterial recanalization procedures were not allowed	90-day mRS 0–1: 33% vs. 38%; OR 0.81 (95% CI 0.36–1.85). Mortality: 15.9% vs. 8.8%; OR 1.95 (95% CI 0.56–7.79).	Feasibility demonstrated, but no efficacy signal; early termination and pre-EVT design limit current applicability.
Normobaric hyperoxia (OPENS-2) [68]	Penumbral oxygenation before and during reperfusion	Multicenter, randomized, single-blind, sham-controlled trial; *n* = 282	Anterior-circulation LVO within 6 h; normobaric hyperoxia initiated before and continued during EVT	90-day median mRS: 2 (IQR 1–4) vs. 3 (IQR 1–4); adjusted cOR 1.65 (95% CI 1.09–2.50); *p* = 0.018.	Positive multicenter trial; independent geographic replication and confirmation of the optimal oxygen protocol are required.

Note: Effect estimates refer to the prespecified primary efficacy outcome unless otherwise stated. For dose-finding or safety-focused studies (APRIL and RHAPSODY), clinical or imaging findings are explicitly identified as secondary or exploratory. Evidence interpretation reflects trial design, statistical precision, directness to contemporary reperfusion practice, and external validity; it is not a formal GRADE or Oxford evidence rating. Abbreviations: 3K3A-APC, 3K3A-activated protein C; AIS, acute ischemic stroke; cOR, common odds ratio; CI, confidence interval; EVT, endovascular thrombectomy; HR, hazard ratio; ICH, intracranial hemorrhage; IQR, interquartile range; IVT, intravenous thrombolysis; LVO, large-vessel occlusion; mITT, modified intention-to-treat; MMP, matrix metalloproteinase; mRS, modified Rankin Scale; NMDA, N-methyl-D-aspartate; NR2B, N-methyl-D-aspartate receptor subunit 2B; OR, odds ratio; PAR1, protease-activated receptor 1; PSD-95, postsynaptic density protein 95; RCT, randomized controlled trial; RR, risk ratio; S1P, sphingosine-1-phosphate; SUR1-TRPM4, sulfonylurea receptor 1–transient receptor potential melastatin 4; TLR4, Toll-like receptor 4.

**Table 2:** Candidate Biomarkers and Phenotypes for Precision Adjunctive Therapy: Intended Uses and Evidence Maturity.

Candidate Biomarker/Phenotype	Biological Relevance	Current Intended Use	Evidence Maturity	Key Requirements for Trial Readiness
Core–penumbra mismatch [88,89]	Salvageable tissue and the pace of infarct progression	Enrollment enrichment; patient stratification	Moderate–high	Harmonize thresholds, software platforms, and definitions across late-window and large-core populations.
Collateral status/HIR [88,89]	Cerebrovascular reserve and severity of hypoperfusion	Prognostication; candidate treatment-effect modifier	Moderate	Prespecify interaction analyses and validate effect modification in randomized trials.
Post-thrombectomy perfusion deficit/no-reflow [52,53,54,55]	Microvascular reperfusion failure despite macrovascular recanalization	Mechanistic endpoint; enrichment for microcirculation-targeted therapy	Low–moderate	Standardize imaging timing, quantitative thresholds, and definitions across modalities.
BBB permeability/edema phenotype [31,32,33,34,89]	Barrier failure, edema propensity, and hemorrhagic risk	Safety stratification; enrichment for BBB- or edema-targeted therapy	Moderate	Demonstrate prediction of differential treatment benefit rather than risk prediction alone.
MMP-9 [90,94]	Basement-membrane degradation and BBB disruption	Prognostication; candidate safety or pharmacodynamic marker	Moderate	Standardize the sampling compartment, temporal profile, assay platform, and decision threshold.
NfL/GFAP [90,91,92,93]	Axonal and astroglial/BBB-associated injury	Prognostication; quantification of injury burden	Moderate	Define actionable acute-phase thresholds and validate treatment-by-biomarker interactions.
Inflammatory mediators/NLR/complement [19,20,90]	Systemic-to-cerebral immune activation	Prognostication; exploratory endotyping	Low–moderate	Address nonspecificity, temporal dynamics, and infection-related confounding; validate reproducible panels.
Multi-omics/single-cell signatures [19,20,95,96]	Cell-state and pathway-defined endotypes	Discovery; target validation; candidate-panel development	Low	Shorten turnaround time; achieve external replication; develop deployable panels; establish causal or treatment-effect relevance.

Note: Evidence maturity is a qualitative appraisal integrating reproducibility, analytical and operational standardization, availability within the acute-care workflow, and evidence of treatment-effect interaction; it is not a formal GRADE rating. Most candidates currently have prognostic rather than predictive validity and should not yet be used to select adjunctive therapy outside prospective trials. Abbreviations: BBB, blood–brain barrier; GFAP, glial fibrillary acidic protein; HIR, hypoperfusion intensity ratio; MMP-9, matrix metalloproteinase-9; NfL, neurofilament light chain; NLR, neutrophil-to-lymphocyte ratio.

## Data Availability

No new data were created or analyzed in this study. Data sharing is not applicable to this article.
